# Hypoxia preconditioning protects neuronal cells against traumatic brain injury through stimulation of glucose transport mediated by HIF-1α/GLUTs signaling pathway in rat

**DOI:** 10.1007/s10143-019-01228-8

**Published:** 2020-01-02

**Authors:** Xiaogang Wu, Chunlin Wang, Jinbiao Wang, Meijie Zhu, Yinsheng Yao, Jiachuan Liu

**Affiliations:** Department of Neurosurgery, No. 901 Hospital of the Chinese People’s Liberation Army Logistic Support Force, Hefei, Anhui China

**Keywords:** Traumatic brain injury, Hypoxic preconditioning, Glucose transporter-1(GLUT1), Glucose transporter-3(GLUT3), Hypoxia-inducible factor-1α(HIF-1α)

## Abstract

Hypoxia preconditioning (HPC), a well-established preconditioning model, has been shown to protect the brain against severe hypoxia or ischemia caused by traumatic brain injury (TBI), but the mechanism has not been well elucidated. Anaerobic glycolysis is the major way for neurons to produce energy under cerebral ischemia and hypoxia after TBI, and it requires large amounts of glucose. We hypothesized that glucose transport, as a rate-limiting step of glucose metabolism, may play key roles in the neuroprotective effects of HPC on cerebral cortex tissue against TBI. The aim of this study was to investigate the effect of HPC on glucose transport activity of rat cerebral cortex tissue after TBI through examining the gene expression of two major glucose transporters (GLUT1 and GLUT3) and their upstream target gene hypoxia-inducible factor-1α (HIF-1α). Sprague-Dawley rats were treated with HPC (50.47 kPa, 3 h/d, 3d). Twenty-four hours after the last treatment, the rats were injured using the Feeney free falling model. Cortex tissues of injured rats were removed at 1 h, 4 h, 8 h, 12 h, 1 day, 3 days, 7 d, and 14 days post-injury for histological analysis. Compared with TBI alone, HPC before TBI resulted in the expression of HIF-1α, GLUT1, and GLUT3 to increase at 1 h; they were markedly increased at 4 h, 8 h, 12 h, 1 day, and 3 days and decreased thereafter (*p* < 0.05). HPC before TBI could improve neuronal survival in rats by examining NeuN staining and observing reduced apoptosis by examining TUNEL staining. The result showed that HPC before TBI could increase the expression of GLUT1 and GLUT3. And through double immunofluorescence staining for GLUT3 and NeuN, the results strongly suggest that HPC improved glucose transport activity of neurons in rats with TBI. In summary, our results further support that HPC can improve hypoxia tolerance and attenuate neuronal loss of cerebral cortex in rats after TBI. The mechanism is mainly related to the increase of glucose transport activity through inducing GLUT1 and GLUT3 expression through upregulating HIF-1α expression.

## Introduction

In recent years, many studies have shown that hypoxia preconditioning (HPC) could increase the resistance of brain to subsequent hypoxic insults [1–3]. Gidday et al. [4] showed that after pre-exposure to 8% oxygen for 3 h for 1 day, the rat brain can be protected from hypoxic-ischemic injury induced 1 day after HPC. On the other hand, the exact neuroprotective mechanisms have not been entirely clarified. Previous studies showed that hypoxia-inducible factor-1α (HIF-1α), a transcription factor closely related to the oxygen concentration in vivo, was the key of the puzzle [5–7]. The regulation of HIF-1α and its target genes has provided a framework for beginning to understand the neuroprotective mechanism of HPC [8, 9].

We previously demonstrated in vivo that cerebral hypoxia-ischemia caused by traumatic brain injury (TBI) plays a crucial role in producing a variety of severe secondary brain damage [10–12]. Moreover, cerebral hypoxia-ischemia after TBI reduces oxygen delivery and leads to disordered glucose metabolism [13, 14]. As a result, the shortage of energy supply is one of the most important causes for neuronal apoptosis. As the most critical protein for glucose transport and which has been proven to be the rate-limiting step of glucose metabolism [15], the glucose transporter (GLUT) plays an indispensable role for energy production to the brain after injury [16]. Fourteen isoforms of GLUTs have been found in mammalian up to now, among which GLUT1 and GLUT3 are predominantly expressed in the brain [17]. The role of GLUT1 is mainly to transport glucose across the endothelial cells of the blood-brain barrier (BBB) and help glucose pass through the glial cell membrane [7]. GLUT3 mainly helps glucose to pass through the neuronal cell membrane [18]. GLUT1 and GLUT3 levels in brain tissue are increased after TBI, strongly suggesting that inhibiting the function of GLUTs leads to abnormal brain function and neuronal death [19]. Studies using different methodologies showed increased glucose metabolism in the affected brain after TBI [20–22]. Higher consumption of glucose will require higher glucose transport. Because only glucose can be used by neurons under anaerobic conditions, increased HIF-1α expression under hypoxia will increase the expression of GLUT1 and GLUT3 to provide supplementary glucose for glycolysis [23–25].

The aim of the present study was to investigate the effects of HPC on glucose metabolism in rat cerebral cortex tissue after TBI. Detailed molecular mechanisms were discussed.

## Materials and methods

### Experimental animals

A total of 204 healthy male SD rats weighing 220–250 g were supplied by the experimental animal center of Anhui Medical University. The experimental procedures were approved by the Animal Care and Use Ethics Committee according to internationally accepted ethical standards. All the rats were housed in a temperature-controlled (22 ± 2 °C) environment with 60% humidity under diurnal lighting condition on a standard 12/12 h light/dark cycle. The rats were randomly divided into the sham group (*n* = 12), the traumatic brain injury group (TBI group, *n* = 96), and the traumatic brain injury after HPC group (HPCT group, n = 96). The rats in the TBI group and the HPCT group were further divided into the subgroups of 1 h, 4 h, 8 h, 12 h, 1 day, 3 days, 7 days, and 14 days randomly after injury, respectively. Each subgroup had 12 rats.

### Traumatic brain injury

**Fig. 1:**
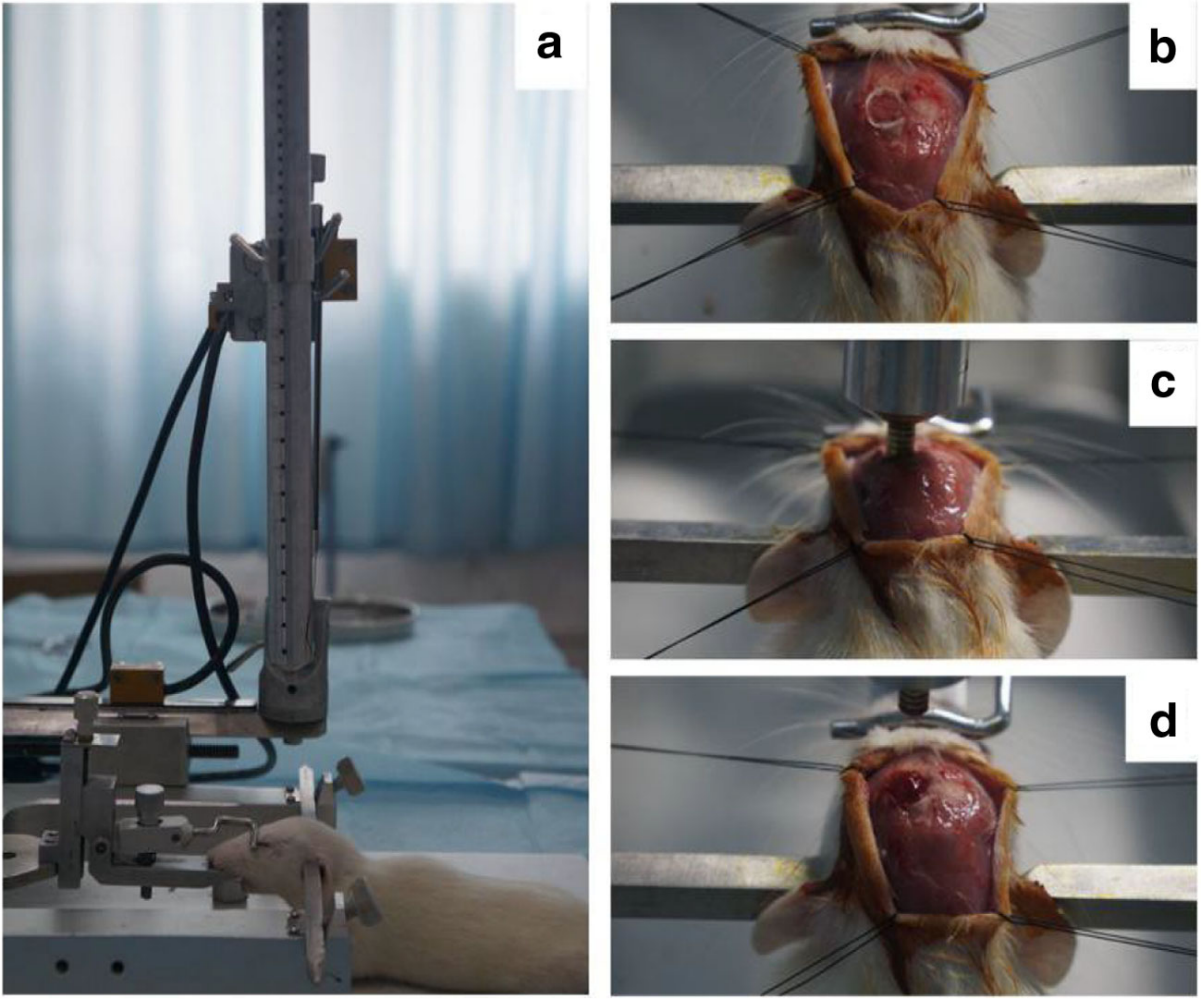
**A** The impact device for the modified free-fall injury. The rat is fixed through its bilateral external auditory canal. **B** Pre-impact state of rats. Part of the left parietal skull and meninges have been dissected, and the brain tissue has been exposed. **C** Rats just before impact. The brain tissue was impacted by a 40-g weight impactor. **D** Post-impact state of rats. The bruising of the brain was obvious and accompanied by hemorrhage

The modified free-fall injury method was used to establish the rat models of TBI, as previously described [26]. After anesthesia with 10% chloral hydrate (0.5 g/kg) by intraperitoneal injection, the rat’s head was fixed in an impact device consisting of a stereotactic frame, a vertical guide tube, and a dropping weight impactor (bottom diameter of 3 mm). A midline incision on the scalp was made to expose the skull. The periosteum was separated. A surgical cranial abrasive drill was used to conduct a left craniotomy with a diameter of 4.0 mm at 0.5 mm from the bregma. The dura mater was kept intact during this operation. A 40-g weight impactor freely fell from a height of 25 cm along the vertical tube to cause brain damage with a 1.0 mm impact depth and a diameter of 5 mm cortical injury area. The scalp was sutured. The rats were provided with ample water and food (Fig. 1).

### Hypoxic preconditioning

**Fig. 2:**
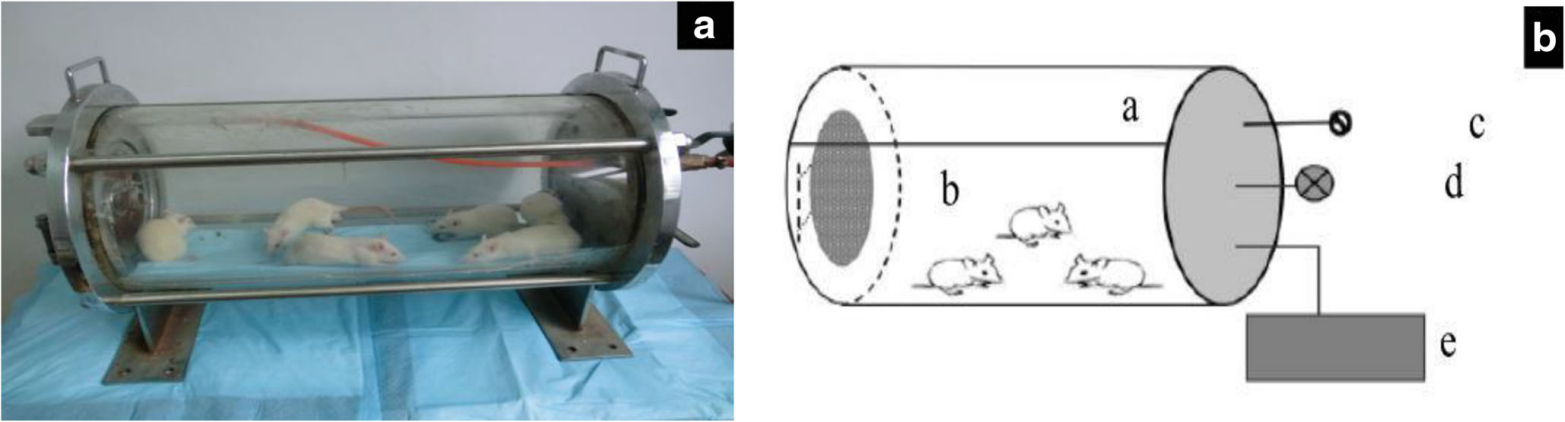
**A** The hypobaric hypoxic chamber model. **B** Schematic of the hypobaric hypoxic chamber. (a) Closed chamber (radius, 15 cm; length, 65 cm); (b) rat access channel; (c) air inlet valves; (d) manometer (range, 0–760 Torr); and (e) vacuum pump. Twelve rats were placed in the closed chamber after acclimatizing to the environment for 5 min. Then, the vacuum pump was connected, and the chamber pressure was decreased to 360 Torr for 3 h

The rats in the HPCT group were preconditioned by intermittent stays in a hypobaric hypoxic chamber (25 °C, 360 Torr) for 3 h/day for 3 days. The percentage of oxygen in the air inductioned by HPC is about 10.4%. The hypobaric hypoxic chamber includes an air inlet valve and a vacuum pump (Fig. 2A). When the rats became familiar with the environment (after 5 min), the vacuum pump was started, and a hypoxic environment of 360 Torr was gradually created. The pressure was maintained for 3 h by regulating the air inlet valves and then returned to the normal room condition. The rats received 3 consecutive days of hypoxia treatment before traumatic brain injury (Fig. 2B).

### Neurologic deficit score

The functional status of the rats was evaluated according to the modified neurological severity score (mNSS) [27] by an observer unaware of grouping. This score is an 18-point scale assessing the functional neurologic status based on motor, sensory, reflex, and behavioral tasks such as beam walking, beam balance, and spontaneous locomotion. The scores were 0 points, no dysfunctions; 1–6 points, mild damage; 7–12 points, moderate damage; and 13–18 points, severe damage (Table 1).

**Table 1:** Modified neurological severity score points

Motor tests	
Raising rat by tail	3
Flexion of forelimb	1
Flexion of hindlimb	1
Head moved 10° to vertical axis within 30 s	1
Placing rat on floor (normal 0; maximum 3)	3
Normal walk	0
Inability to walk straight	1
Circling toward paretic side	2
Falls down to paretic side	3
Sensory tests	2
Placing test (visual and tactile test)	1
Proprioceptive test (deep sensation, pushing paw against table edge to stimulate limb muscles)	1
Beam balance tests (normal 0; maximum 6)	6
Balances with steady posture	0
Grasps side of beam	1
Hugs beam and 1 limb falls down from beam	2
Hugs beam and 2 limbs fall down from beam, or spins on beam (60 s)	3
Attempts to balance on beam but falls off (40 s)	4
Attempts to balance on beam but falls off (20 s)	5
Falls off; no attempt to balance or hang on to beam (20 s)	6
Reflex absence and abnormal movements	4
Pinna reflex (head shake when auditory meatus is touched)	1
Corneal reflex (eye blink when cornea is lightly touched with cotton)	1
Startle reflex (motor response to a brief noise from snapping a clipboard paper)	1
Seizures, myoclonus, myodystony	1
Maximum points	18

### Histology

The rats were sacrificed by cervical dislocation after behavioral testing. The dura mater was removed, and brain tissues were collected at 0 °C at 1 h, 4 h, 8 h, 12 h, 1 day, 3 days, 7 days, or 14 days after injury. Each subgroup consisted of 12 rats. For quantitative real-time PCR and western blot analyses, six of the rats were only infused with 0.9% normal saline (NS), the brain tissues were quickly removed, and the pericontusional cortex (5 mm around the round injury, 3 mm deep, and after removing the injured area) was collected and stored in liquid nitrogen (−80 °C) until use. For immunohistochemistry and immunofluorescence, the other six rats in each subgroup were sacrificed and perfused intracardially with normal saline and fixed with 500 mL of 4% paraformaldehyde. The brain tissues were quickly removed and fixed with 4% paraformaldehyde, embedded in paraffin, and sectioned. The sections (4 μm thick) were stained with hematoxylin and eosin. The tail side of each slice was selected for image analysis with pathological image analysis system (Sigma Scan Pro5.0 software), and the lesion area and total area of each slice were measured. The lesion volume of each slice was the product of lesion area and thickness of that slice, and the sum of lesion volume of each slice was considered as the total lesion volume.

### Cerebral edema evaluation

Cerebral edema formation was examined using a wet/dry method [28] at 1 day after injury. Bilateral brains were removed and weighted immediately, dried for 24 h in a 100 °C oven (*n* = 6 rats/group), and weighed again as dry weight. The water content of the tissue was calculated as:

H_2_O = (wet weight-dry weight)/wet weight×100%.

### Quantitative real-time polymerase chain reaction (qRT-PCR)

Total RNA was extracted from the pericontusional area using the Ultrapure RNA Kit (TIANGEN, Beijing, China), following the manufacturer’s instructions. RNA was reverse-transcribed to complementary DNA (cDNA) using the RevertAid First Strand cDNA Synthesis Kit (Thermo Fisher, MA, USA). Primers for each transcript were synthesized by Invitrogen (Shanghai, China) and are listed in Table 2. GAPDH was used as endogenous controls for GLUT-1, GLUT-3, and HIF-1α. The SYBR®Green Real-time PCR Kit (Takara, Liaoning, China) was used for qRT-PCR assays using the ABI 7500 Real-Time PCR System (Applied Biosystems, CA, USA). Relative expression was normalized to that of endogenous controls using the comparative cycle threshold method, and the fold change in gene expression was calculated using the 2^-∆∆^Ct method.

**Table 2:** cDNA of HIF-1α, GLUT-1, GLUT-3, and GAPDH

Gene	Primer sequences(5^′^-3^′^)	Product
HIF-1α	Sense: AGAGTCAAGCCCAGAGTCAC	116 bp
	Antisense: TGGGACTGTTAGGCTCAGGT	
GLUT-1	Sense: TTATTGCCCAGGTGTTCGGC	93 bp
	Antisense: GTAGCAGGGCTGGGATGAAG	
GLUT-3	Sense: TGTGGCTCAGGTCTTTGGTT	99 bp
	Antisense: GCGCTCTGTAGGATAGCTGG	
GAPDH	Sense: GGGCTCTCTGCTCCTCCCTGT	107 bp
	Antisense: ACGGCCAAATCCGTTCACACC	

### Western blot

Tissue samples from the pericontusional area were washed with cold PBS and lysed in ice-cold RIPA buffer (1 ml per 100 mg tissue sample) containing protease inhibitors (Beyotime, China), followed by incubation on ice for 5 min and centrifugation at 4 °C for 15 min. The supernatant was collected, and protein concentration was measured with BCA (Beyotime, China). Equal amounts of protein from each sample were separated by electrophoresis on a 10% SDS-polyacrylamide gel (Beyotime, China), electrotransferred to a PVDF membrane (Millipore, USA), and blocked with 5% skimmed milk powder. Rabbit anti-β-actin polyclonal antibody (1:1000, Abcam Biotechnology, UK), rabbit anti-HIF-1α polyclonal antibody (1:200, Abcam Biotechnology, UK), rabbit anti-GLUT-1 polyclonal antibody (1:200, Abcam Biotechnology, UK), and rabbit anti-GLUT-3 polyclonal antibody (1:200, Abcam Biotechnology, UK) were used. Immunoblots were visualized by chemiluminescence using an ECL detection system (Thermo Fisher, MA, USA). The intensity of the bands was determined using the Quantity One 4.6.2 software.

### Immunohistochemistry

Sections of brain tissue were incubated with 3% H_2_O_2_ at 37 °C for 10 min, in normal goat serum for 30 min at room temperature, and overnight at 4 °C with 100 μL of rabbit serum and primary antibodies of interest overnight and then rinsed 3 times with 0.1 M phosphate-buffered saline (PBS). The antibodies were rabbit anti-HIF-1α polyclonal antibody (1:200, Abcam Biotechnology, UK), rabbit anti-GLUT1 polyclonal antibody (1:200, Abcam Biotechnology, UK), rabbit anti-GLUT3 polyclonal antibody (1:200, Abcam Biotechnology, UK), or rabbit anti-NeuN polyclonal antibody (1:200, Abcam Biotechnology, UK). The sections were incubated with secondary antibodies: biotin conjugates and diaminobenzidine from the SP rabbit or PV goat kit (1:1000, ZSGB Biotechnology, China). Hematoxylin was selected as the counterstain. An examiner blinded to the experimental groups detected cells labeled with HIF-1α, GLUT1, GLUT3, or NeuN under a 400× light microscope. The mean absorbance value in randomly selected fields of view was calculated using the JEDA 801D morphologic image analysis system (Molecular Devices, Sunnyvale, CA, USA) and the MetaMorph software (Molecular Devices).

### TUNEL assay

TUNEL staining (Roche, USA) was used for detecting cell apoptosis. The sections were permeabilized with 1% proteinase K (in 50 mM Tris/5 mM EDTA buffer) for 10 min, rinsed with 0.1 M PBS for 5 min, and incubated in a TUNEL reaction mixture for 1 h at 37 °C. The sections were rinsed with PBS for 5 min and visualized by staining with 0.02% 3,3′-diaminobenzidine. An examiner blinded to the groups detected apoptotic cells under a 400× light microscope.

### Double-labeling immunofluorescence staining

To detect the GLUT-3 neurons, double immunofluorescence staining for NeuN (red) and GLUT3 (green) was performed under identical conditions for all sections. The sections were defrosted, fixed with acetone at 4 °C for 30 min, and washed with PBS for 5 min three times. Antigen retrieval was performed by boiling in antigen retrieval solution at 100 °C for 5 min and washed with PBS for 5 min three times. Sections were blocked with bovine serum albumin (Biosharp, Shandong, China) at room temperature for 1 h. When the blocking solution was removed, the sections were incubated with primary antibodies against GLUT3 (rabbit anti-rat antibody; 1:200, Abcam Biotechnology, UK) and neuronal nuclei (NeuN; mouse anti-rat antibody; 1:200, Abcam Biotechnology, UK) at 4 °C overnight. Following three rinses with PBS, the sections were incubated with TRITC-conjugated anti-rabbit IgG (1:500; Sigma-Aldrich) and FITC-conjugated anti-mouse IgG (1:200; Sigma-Aldrich) at room temperature for 1 h in the dark. Nuclear staining was performed with DAPI (OriGene Technologies, Inc.) for 5 min, and sections were washed in the dark with PBS for 5 min three times. Tissues were observed and photographed under a fluorescence microscope at 400× magnification (MTC-600; Bio-Rad Laboratories, Inc., Hercules, CA, USA).

### Statistical analysis

Data are presented as means ± standard error of the mean. Differences among groups at different time points were compared using one-way ANOVA, with the Student-Newman-Keuls post hoc test. *P* values < 0.05 were considered statistically significant. There were 6 animals per group per time point.

## Result

### HPC attenuates neurological deficit and cerebral edema

**Fig. 3:**
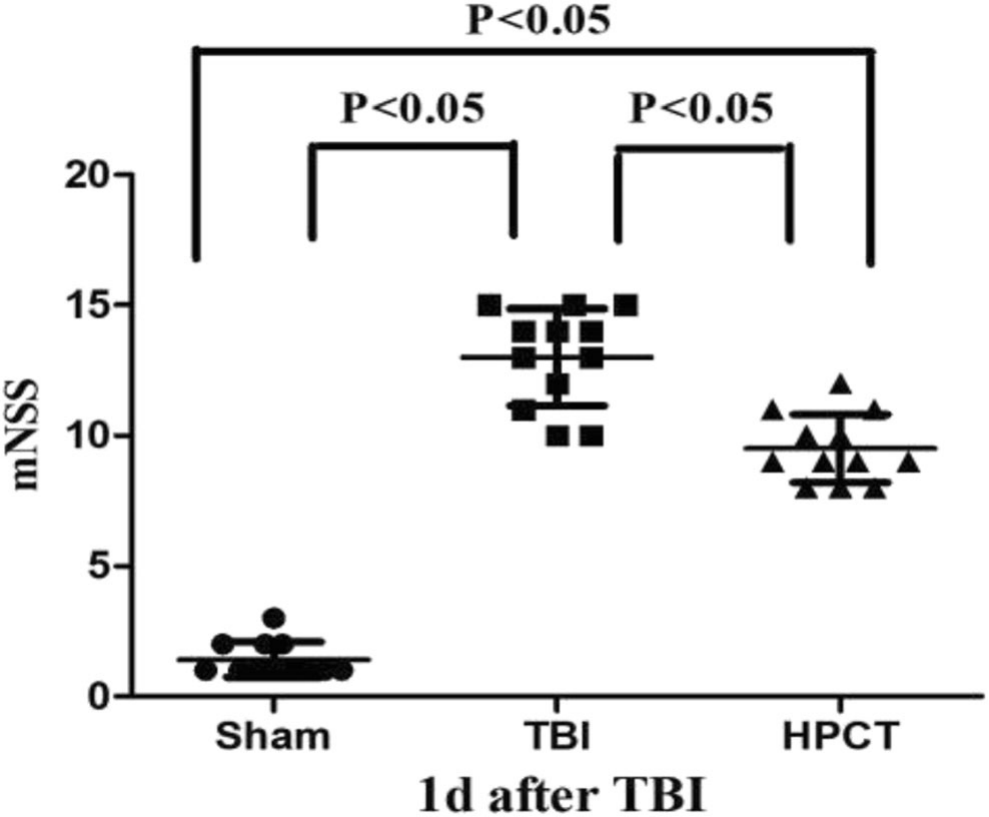
Neurological function was measured by mNSS tests at 24 h after TBI. Compared with the TBI group, the neurological severity scores in the HPCT group were significantly reduced (10.35 ± 1.16 vs. 13.29 ± 2.05, *p* < 0.05, F = 22.57). Data are expressed as means ± SD

Neurological function was examined using the mNSS test at 1 day post-injury in all rats. Rats in TBI group showed higher scores than the sham group (13.29 ± 2.05 vs. 2.16 ± 0.19, *p* < 0.05,F = 19.34). Compared with the TBI group, the neurological severity scores were significantly reduced in the HPCT group (10.35 ± 1.16 vs. 13.29 ± 2.05, *p* < 0.05, F = 22.57) (Fig. 3). According to the mNSS, the injury has to be considered moderate or severe in TBI rats and mostly moderate in HPCT rats.

**Fig. 4:**
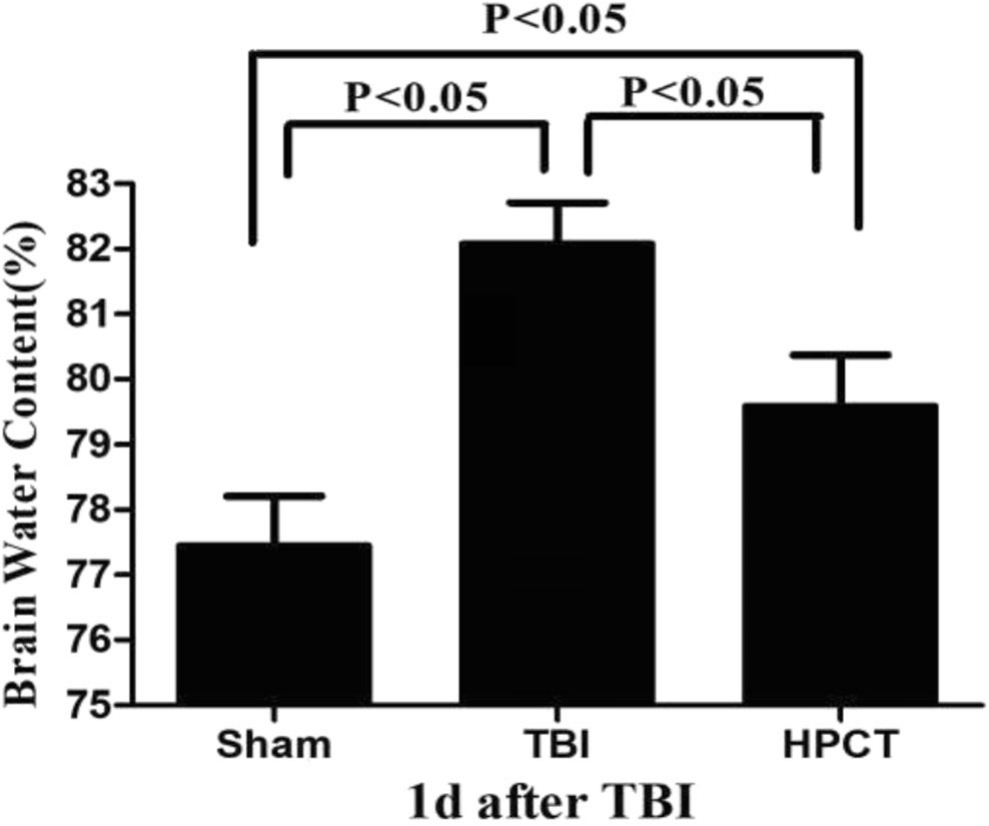
Edema formation was determined at 1 day after TBI. HPC reduced post-injury brain edema. Compared with the TBI group, the neurological severity scores in the HPCT group was significantly reduced (79.23% ± 0.66% vs. 81.79% ± 0.95%, *p* < 0.05, F = 69.47). Data are expressed as means ± SD (*n* = 6 animals per group)

Brain water content is an important prognostic indicator after TBI. The brain water content at 1 day after injury is shown in Fig. 4. The brain water content was increased in the TBI group compared with the sham group (81.79% ± 0.95% vs. 77.34% ± 0.66%, *p* < 0.05, F = 58.39). HPC caused a significant decline in brain water content in the area around the contusion after TBI (79.23% ± 0.66% vs. 81.79% ± 0.95%, *p* < 0.05, F = 69.47) (Fig. 4).

### HPC reduces lesion volume in rats following TBI

**Fig. 5:**
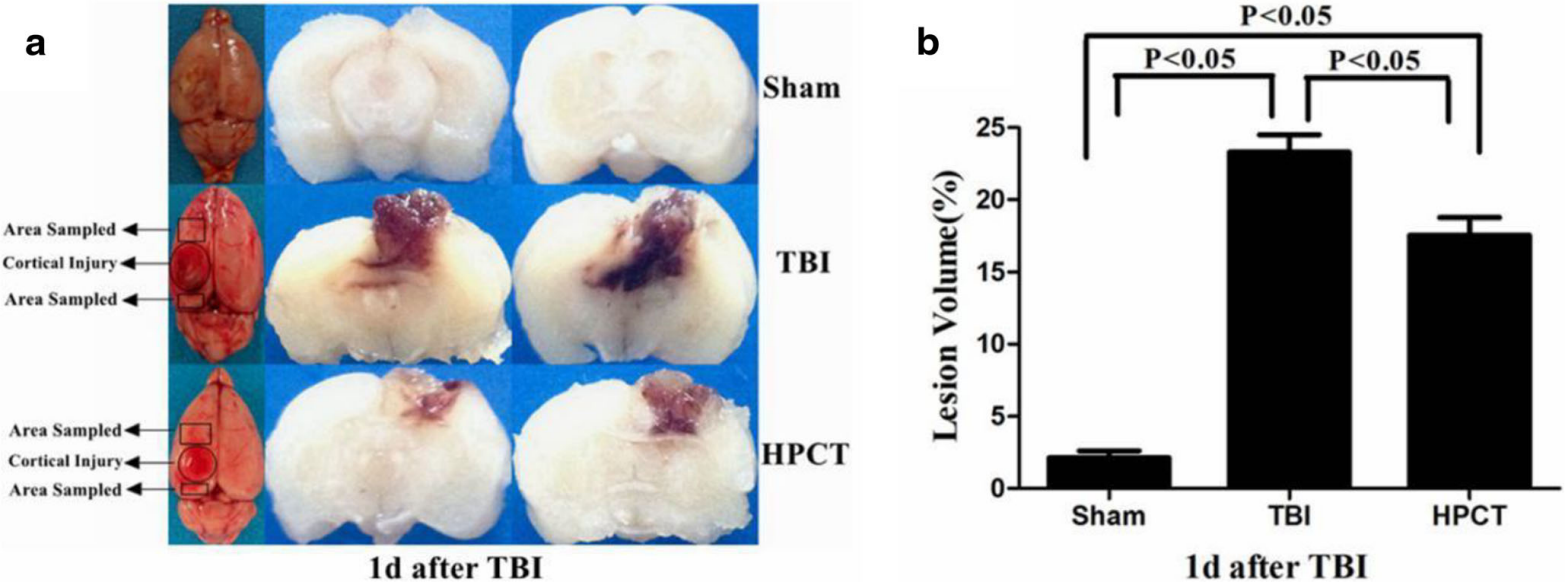
Effects of HPC on brain tissues of rats 1 days post-injury. **A** Representative images of the contusion area induced by the traumatic injury and the sampled area around the injured brain. **B** Quantification of the contusion area at 1 day after injury (% of contralateral hemisphere). The results revealed that TBI caused contusion in the cerebral cortex (22.49% ± 0.19% vs. 2.03% ± 0.03%, *p* < 0.05, F = 19.83). HPC significantly decreased contusion in the cerebral cortex in the HPCT group compared with the TBI group (16.73% ± 0.11% vs. 22.49% ± 0.19%, *p* < 0.05, F = 23.49). Data are expressed as means ± SD (*n* = 6 animals per group)

Twenty-four hours after injury, lesion volume was calculated and shown in Fig. 5. Compared with Sham rats, TBI caused contusion in the cerebral cortex (22.49% ± 0.19% vs. 2.03% ± 0.03%, *p* < 0.05, F = 19.83). Lesion volume was smaller in the HBCT group compared with the TBI group (16.73% ± 0.11% vs. 22.49% ± 0.19%, *p* < 0.05,F = 23.49) (Fig. 5).

### Time course of HIF-1α, GLUT1, and GLUT3 mRNA expression in the injured pericontusional cortex

**Fig. 6:**
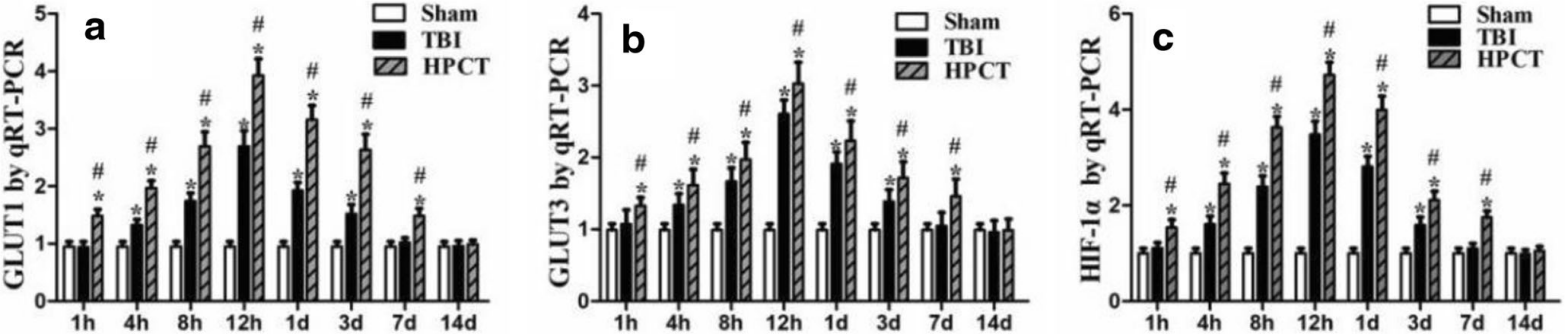
Quantitative real-time PCR analysis of the mRNA levels of GLUT1, GLUT3, and HIF-1α in the cortex after TBI. The relative mRNA expression of GLUT1 (A), GLUT3 (B), and HIF-1α (C) was evaluated by determining the ratio of the expression of the target mRNA to the GAPDH mRNA. The bar graphs show that the mRNA expressions of GLUT1, GLUT3, and HIF-1α were significantly increased in the HPCT group. Data are expressed as means ± SD (*n* = 6 animals per group); *p < 0.05 vs. Sham group, #*p* < 0.05 vs. TBI group

Quantitative real-time PCR was performed to determine the mRNA expression of GLUT1 and GLUT3 in the sham group and in rats sacrificed at 1 h, 4 h, 8 h, 12 h, 1 day, 3 days, 7 days, and 14 days after injury. Compared with TBI alone, HPC before TBI resulted in increased mRNA expression of GLUT1 and GLUT3 as early as 1 h, peaking at 12 h and being markedly increased at 4 h, 8 h, 12 h, 1 day, and 3 days). GLUT1 and GLUT3 mRNA levels in the HPCT group approached the levels observed in the sham group but were still significantly different at 7 days after TBI (TBI group vs. Sham group, *p* < 0.05; HPCT group vs. TBI group, *p* < 0.05) (Fig. 6A-B).

We also detected the mRNA expression of the HIF-1α by quantitative real-time PCR. HIF-1α mRNA was barely detectable in the sham group but was higher in the TBI group at 4 h, 8 h, 12 h, 1 day, and 3 days (*p* < 0.05). In the HPCT group, the mRNA levels of HIF-1α began to increase at 1 h and were markedly increased at each time point (4 h, 8 h, 12 h, 1 d, and 3 days) and were sustained at these elevated levels until 7 days after injury (*p* < 0.05) (Fig. 6C).

### Time course of HIF-1α, GLUT1, and GLUT3 protein expression in the injured pericontusional cortex

**Fig. 7:**
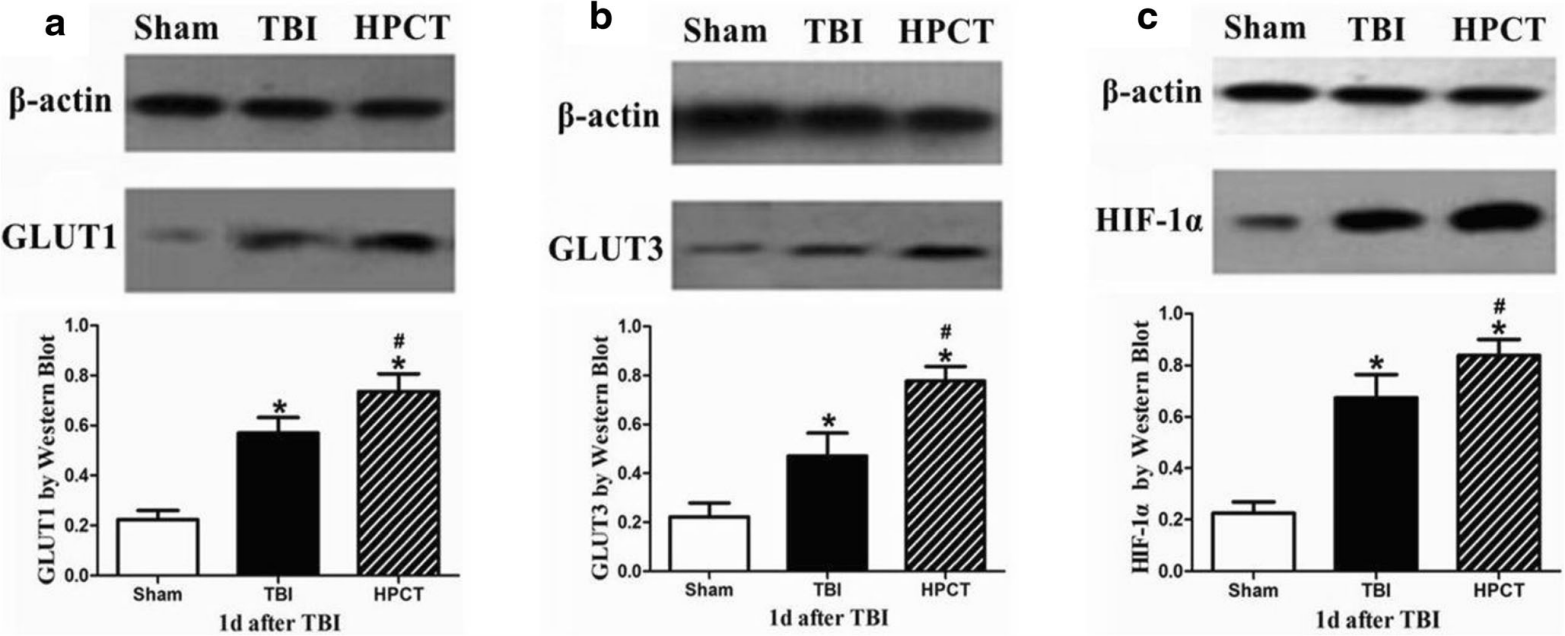
Expression of the GLUT1, GLUT3, and HIF-1α proteins in the pericontusional cortex. Electrophoretic band photographs of the western blot analysis of GLUT1, GLUT3, HIF-1α, and β-actin proteins in the cortex. As shown in the graphs, TBI caused an increase in protein expression of GLUT1 (A), GLUT3 (B), and HIF-1α (C) compared with those in the sham group (*n* = 6 animals per group,*p < 0.05), and HPC increased the expressions of GLUT1 (A), GLUT3 (B), and HIF-1α (C) proteins after TBI. Data are expressed as means ± SD (n = 6 animals per group, ^#^*p* < 0.05)

To examine the effect of HPC on HIF-1α, GLUT1, and GLUT3 protein expression, we measured their levels in cerebral cortex tissues around the injury at 1 day after injury by western blot. As shown in Fig. 7, TBI caused an increase in protein expression of GLUT1 and GLUT3 compared with those in the sham group (Fig. 7A, B). HPC before injury resulted in markedly increased protein levels of both GLUT1 and GLUT3 at 1 day after injury (*p* < 0.05) (Fig. 7A, B).

The protein expression of the HIF-1α was also detected by western blot. Similar to the mRNA levels, the results indicated that the HIF-1α protein was barely detectable in the sham group but were significantly increased in the TBI group. Moreover, the expression of HIF-1α in the HPCT group increased significantly compared with TBI group (*p* < 0.05) (Fig. 7C).

### HPC elevates the expression of HIF-1α, GLUT1, and GLUT3 in pericontusional cortex after TBI

**Fig. 8:**
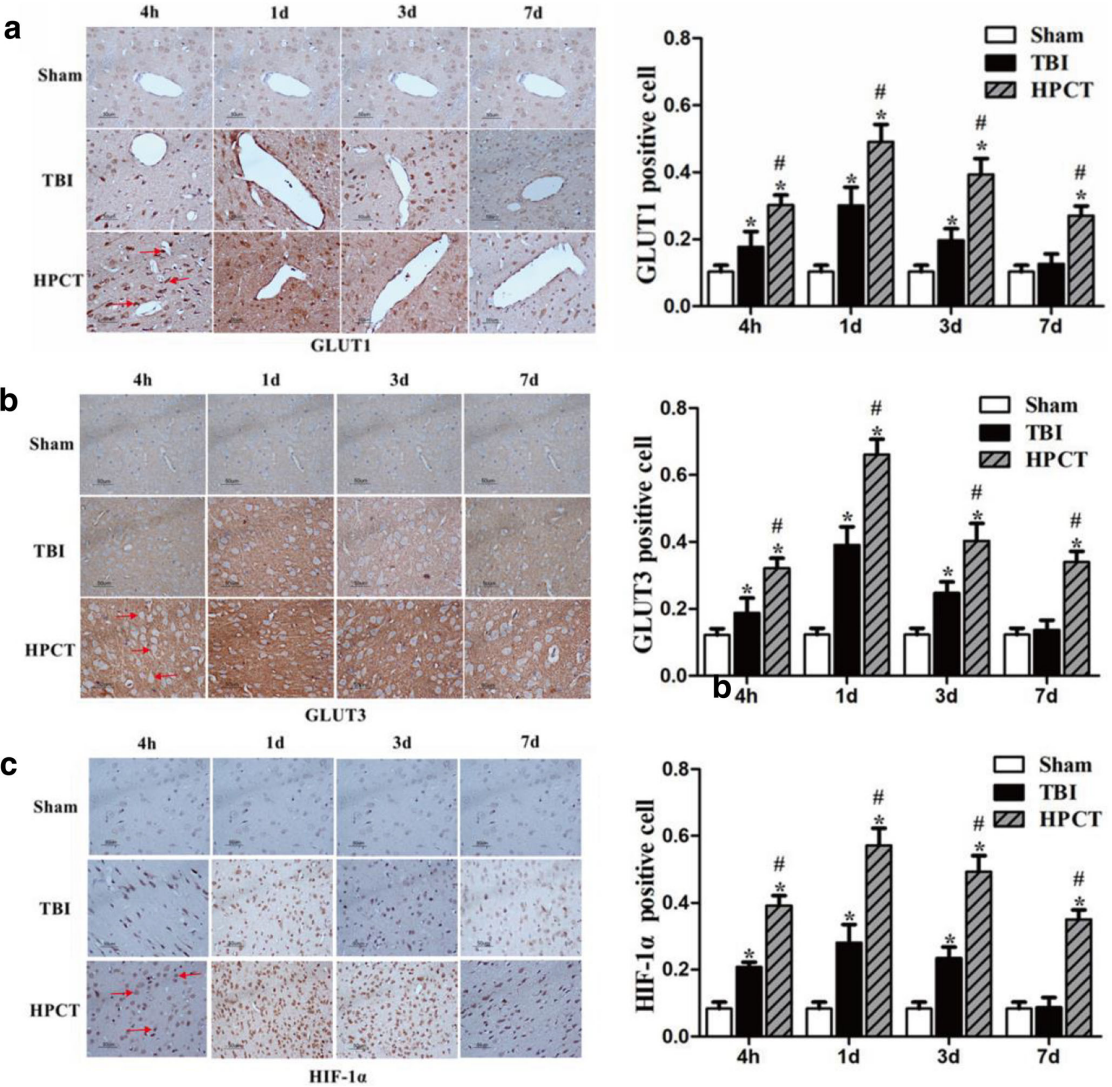
Effect of HPC on the expressions of GLUT1, GLUT3, and HIF-1α in the pericontusional cortex after injury by immunohistochemistry. Scale bar = 50 μm, 400× magnification, red arrows indicate positive stained cells. The bar graphs illustrate that the expressions of GLUT1 (A), GLUT3 (B), and HIF-1α (C) were significantly higher in the HPCT group. Data are expressed as means ± SD (*n* = 6 animals per group). ^*^p < 0.05 vs. Sham group, ^#^*p* < 0.05 vs. TBI group

GLUT1 and GLUT3 expression was assessed by immunohistochemistry. GLUT1 immunostaining was observed throughout the cortex in samples from the TBI group, which was characterized by a relatively strong staining of the wall of the blood vessels. Marked enhancement of GLUT1 immunoreactivity was observed in the HPCT group compared with TBI group (*p* < 0.05). We also observed extensive positive immunostaining of GLUT3 throughout neurons from the TBI group. The enhancement of GLUT3 immunoreactivity in neurons was significantly higher in the HPCT group than in the TBI group (*p* < 0.05) (Fig. 8A, B).

The sham group showed very weak HIF-1α immunostaining, but the levels of HIF-1α were upregulated at 4 h, 1 day, and 3 days (*p* < 0.05). Compared with the TBI group, the expression of HIF-1α in the HPCT group increased significantly and peaked at 1 day after injury (*p* < 0.05) (Fig. 8C).

### HPC improves neuronal survival and reduces apoptosis in pericontusional cortex after TBI

**Fig. 9:**
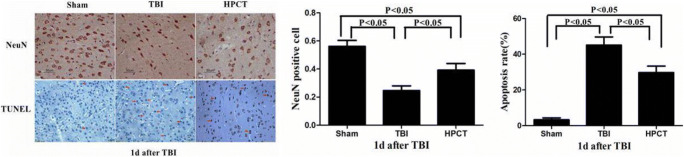
Immunohistochemistry of NeuN and TUNEL in pericontusional cortex of rats at 1 day after injury. Scale bar = 50 μm, 400× magnification, red arrows indicate positive stained cells. The bar graphs show that the expression of NeuN was significantly increased in the HPCT group, whereas the apoptotic rate was significantly reduced in the HPCT group in comparison with the TBI group. Data are expressed as means ± SD (*n* = 6 animals per group)

In order to observe neuronal survival and apoptosis after injury, we used immunohistochemistry to detect the expression of NeuN and TUNEL at 1 day after injury. NeuN-positive cells in the sham group were oval or circular, evenly distributed and regular in morphology, while several NeuN-positive cell nuclei were seen in the sham group. TBI resulted in a mean loss of NeuN staining in the area around the cortical contusion in the TBI group, and part of neuron cytoplasms were reduced and showed agglutination and vacuoles (*p* < 0.05). The number of brown-stained NeuN-positive cell nuclei was significantly increased (*p* < 0.05). A significant gain of NeuN staining and decrease of TUNEL staining displayed a larger number of surviving neurons and fewer apoptosis in the HPCT group compared with the TBI group (*p* < 0.05). Our results indicate that HPC enhanced the survival of neurons and reduced apoptosis in rats after TBI (Fig. 9).

### HPC upregulates GLUT3 co-localization with neurons in the injured pericontusional cortex

**Fig. 10:**
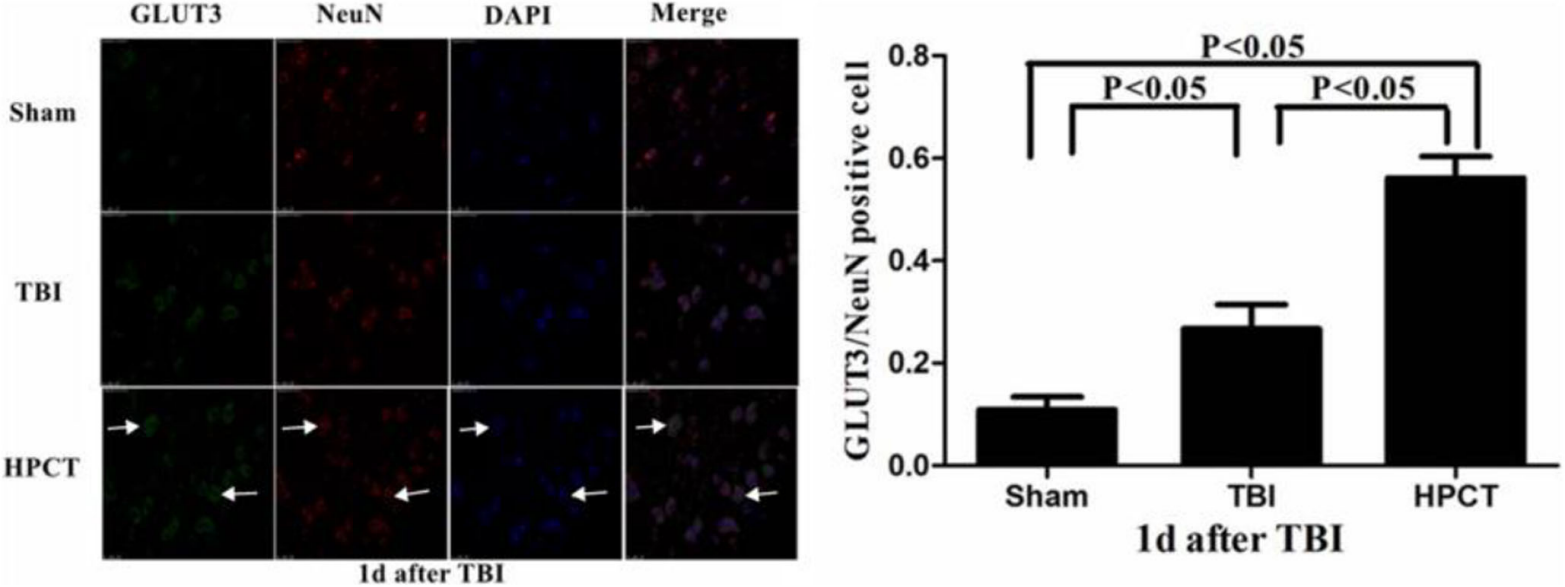
Alterations in GLUT3 expression in neurons of the injured pericontusional cortex at 1 day after TBI (400× magnification). Positive co-expression of NeuN (red) and GLUT3 (green) was observed in the same cells. GLUT3 was expressed in the cytoplasm, whereas NeuN was predominantly expressed in the nuclei. White arrows indicate positive-stained cells. The bar graph illustrates that the expression of GLUT/NeuN co-labeling cells was significantly increased in the HPCT group. Data are expressed as means ± SD (*n* = 6 animals per group)

Double immunofluorescence staining for GLUT3 and NeuN was carried out to evaluate the role of HPC on the expression of GLUT in the neurons in the injured pericontusional cortex. Immunostaining found co-labeling cells when GLUT3 and NeuN were merged around the injured cerebral cortex tissues of the rats in the 24-h subgroup. The number of GLUT/NeuN co-labeling cells in the HPCT group was higher than in the TBI group (*p* < 0.05). These results strongly suggest that HPC improved glucose transport activity of neurons in rats in response to TBI (Fig. 10).

## Discussion

Energy metabolic disorders of brain tissues are mainly due to ischemia and hypoxia caused by TBI, and it is one of the main reasons for neuronal apoptosis and neurological sequelae after TBI [29]. In this study, we provide significant evidence that HPC increases the glucose transport activity of brain tissues after TBI. The levels of GLUT1 and GLUT3 mRNA and protein were also increased. Nevertheless, the exact mechanisms for the increased glucose transport activity of HPC before TBI remain not well understood.

TBI is an important clinical problem worldwide, but its treatment had no major breakthroughs recently to reduce mortality and improve functional outcome. TBI is associated with significant neurological impairments [30, 31], as observed in the rats of the present study. All TBI rats had moderate to severe symptoms, while HPCT rats had moderate symptoms, suggesting that HPC alleviated brain injury, as supported by previous studies [2, 12].

The HIF-1 pathway-mediated gene expression may perform a major function in hypoxia-induced tolerance [32]. It raises a high possibility that HIF-1 may mediate the upregulation of GLUT1 and GLUT3 mRNAs in the HPCT brain tissues. HIF-1, a transcriptional inducer highly sensitive to oxygen concentration, is composed of 120 kD α subunits and 91–94 kD β subunits, in which HIF-1α is the active subunit of oxygen regulation [33]. The expression and activity of HIF-1α are very low under normal oxygen conditions. In hypoxia, the expression of HIF-1α increases, which regulates the expression of downstream target genes [34]. HIF-1α exposure can promote the expression of GLUT-3 mRNA through the PI3K/Akt/mTOR pathway [35]. In this study, the expression of HIF-1α mRNA and proteins increased at 1 h after injury in the HPCT group and increased significantly more than in the TBI group from 4 h to 7 days after injury compared to the TBI group. The present study showed that HPC may increase the tolerance of brain tissue to hypoxia by increasing the expression of HIF-1α early after injury and promote the continuous high expression of HIF-1α within 7 days after injury to facilitate the regulation of changes in the expression of its target genes and to play the role of brain protection.

After TBI, the affected area may suffer from hypoxia because of structural damage and edema [12, 19, 28]. In the present study, the TBI group indeed showed higher water content in the affected area, while the HPCT group showed lower edema. The present study was not designed to determine the cause for lower water retention after HPC and TBI, but lower inflammation might be involved [36]. Nevertheless, hypoxia will lead to increased HIF-1α expression, which will in turn increase the levels of GLUT1 and GLUT3 in order to increase the glucose supply to the hypoxic neurons for glycolysis [23–25]. Indeed, TBI is associated with higher glucose metabolism in the injured area [20–22]. Of course, proper energy supply is conducive to reduce neuronal death, as shown in the present study. Indeed, the TBI groups showed markedly increased neuronal apoptosis, which was decreased in the HPCT group.

Nevertheless, the exact mechanisms for the protective effects of HPC remain elusive. One possibility is the stimulation of GLUT-1 and GLUT-3 mRNAs and protein in brain tissues after HPC [37]. Accordingly, in the present study, we showed the following: (1) the expression of GLUT-1 and GLUT-3 mRNA and protein in the HPCT group began to increase at 1 h after injury compared to the TBI group. The results indicated that HPC can improve the ability of cortical brain tissue to adapt to hypoxia beforehand, so as to facilitate the advanced expression of GLUT-1 and GLUT-3 mRNA and proteins in cortical tissues in the subsequent occurrence of TBI and significantly increase the ability of the brain to transport and absorb glucose at the early stage after injury. (2) The expression of GLUT-1 and GLUT-3 mRNA and proteins in the HPCT group was significantly higher than in the TBI group between 4 h and 3 days after injury, suggesting that HPC can increase the hypoxia tolerance of neurons and improve the ability of neurons to use glucose by increasing the amount of glucose through increasing the expression of GLUT-1 and GLUT-3 in cortical tissues after TBI. The aim is to reduce the energy metabolism disorder of neurons due to insufficient aerobic metabolism after injury and to maintain the relative integrity of the structure and function of neurons. (3) The expression of GLUT-1 and GLUT-3 mRNA and protein in the TBI group was close to that of the Sham group at 7 days after injury but was still higher in the HPCT group than in the TBI group. The results suggest that HPC prolonged the hypoxia tolerance of the injured neurons and prompted neurons to continuously ingest more glucose by maintaining the continuous expression of GLUT-1 and GLUT-3 mRNA and protein after injury. All of the above results demonstrate that HPC can increase glucose transport activity in TBI, possibly through upregulating the expression of glucose transporters.

NeuN is a neuron-specific nuclear protein and is expressed in surviving neuronal cells [38]. The expression of NeuN, as an important indicator to detect neuronal necrosis, decreased or even disappeared after neuronal ischemia and hypoxia [39]. This study suggests that HPC increased the expression of NeuN after injury. By contrast, TUNEL-positive cells were significantly increased after injury and significantly reduced by HPC. Furthermore, NeuN/GLUT-3 immunofluorescence double staining showed that HPC can reduce neuronal damage caused by local energy supply disorders by regulating the expression of glucose transporters.

## Conclusions

In this study, we clearly demonstrated that HPC before TBI can induce hypoxic tolerance in cerebral cortex of rats after TBI, as measured by GLUT-1 and GLUT-3, in conjunction with an increase in HIF-1α mRNA and protein levels. Our findings suggest that HPC improves glucose transport in the TBI-injured brain through enhanced mRNA and protein levels of GLUT1 and GLUT3. GLUT1 and GLUT3 may be downstream targets in the HPC neuroprotective pathway.
